# Seroepidemiological Survey on Bluetongue Virus (BTV) among Cattle, Sheep, and Goats in Gadarif State, Eastern Sudan

**DOI:** 10.1155/2024/7712412

**Published:** 2024-09-18

**Authors:** Hatim H. Abraheem, Mohammed O. Hussien, Amira M. Elhassan, Khalid A. Enan, Azza B. Musa, Selma K. Ahmed, Abdel Rahim M. El Hussein

**Affiliations:** ^1^ Central Veterinary Research Laboratory (CVRL) Animal Resources Research Corporation (ARRC) P.O. Box 8067, El Amarat, Khartoum, Sudan; ^2^ Central Laboratory Ministry of Higher Education and Scientific Research P.O. Box 7099, Khartoum, Sudan

## Abstract

Bluetongue (BT) is an arthropod-borne viral disease that primarily affects ruminants in tropical and temperate regions. In the present study, a cross-sectional survey was conducted to define the seroprevalence of Bluetongue virus and to identify the possible risk factors correlated with BTV seropositivity among cattle, sheep, and goats during the period 2015-2016 in Gadarif State. A total of 420 cattle, 877 sheep, and 641 goat serum samples were collected randomly from 12 localities. Information about age, sex, breed, area ecology, and location was obtained for each sample. Bluetongue seroprevalence was estimated using competitive enzyme-linked immunosorbent assay (cELISA). The overall seroprevalence of BTV was 92.9% (390/420), 76.4% (670/877), and 85.3% (547/641) among cattle, sheep, and goats, respectively. Multivariate analysis followed univariate analysis showed that there was a significant difference (*p* < 0.05) between location, area ecology and age groups of cattle, sheep, and goats, and seropositivity to BTV. In addition, a significant association (*p* < 0.05) was observed between sex and seropositivity to BTV in sheep. In conclusion, BTV antibodies are highly prevalent in Gadarif State and susceptible livestock are at risk of exposition with BTV. Consequently, these animals have protection against specific BTV serotypes.

## 1. Introduction

Bluetongue (BT) is an infectious, noncontagious arthropod-borne disease of ruminants, caused by bluetongue virus (BTV). It is the type species of the genus *Orbivirus* within the virus family *Reoviridae* and possesses a genome consisting of 10 segments of double-stranded RNA (dsRNA) encoding 7 structural and 4 nonstructural proteins. BTV is transmitted between vertebrate hosts via the bites of midges of the genus *Culicoides* [1, 2]. Bluetongue was first reported in sheep in southern Africa in 1881 [3]. BTV naturally infects a wide range of animals including domestic and wild ruminants such as sheep, goat, cattle, buffaloes, deer, most species of African antelope, and camels. Infection with bluetongue virus (BTV) is unapparent in the vast majority of animals but can cause fatal disease in a proportion of infected sheep, deer, and wild ruminants [4]. Twenty-six (26) different serotypes have been identified, and the ability of each strain to cause disease varies considerably. Vaccination is used as the most effective and practical measure to minimize losses related to the disease and to potentially interrupt the cycle from the infected animal to vector along with insect control measures. BT is known to be endemic all over Sudan [5–9]. Little BT studies were conducted in Eastern Sudan including Gadarif. The present investigation aimed to define the seroprevalence of BTV among cattle, sheep, and goats and to identify risk factors correlated with the BTV exposure in Gadarif State.

## 2. Materials and Methods

### 2.1. Study Area

**Figure 1:**
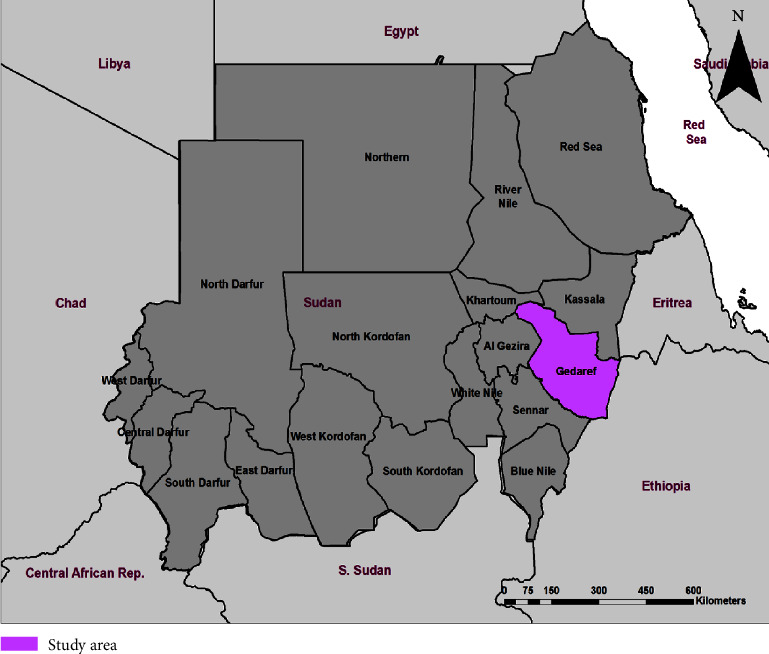
Map of Sudan showing the study area (Gadarif State) where serum samples were collected [https://onlinelibrary.wiley.com/doi/10.1155/2021/6613217] [28].

Gadarif State lies between 16.14° altitudes and 33.35° longitudes and is bordered by five Sudanese states, an international border of Eritrea and Ethiopia. The study area is divided into twelve localities (Figure 1). Based on ecological features, the study area was classified into four groups: desert, semiarid, savanna, and rich savanna.

### 2.2. Study Design

A cross sectional study was conducted to determine the prevalence of antibodies against BTV in cattle, sheep, and goats using the competitive enzyme-linked immunosorbent assay (cELISA). The sample size was calculated according to Thrusfield [10]. Sample size for this survey was estimated using the formula *n*=*zz*^2^*PQ*/*L*^2^, where *n* was the required number of individuals to be examined; *z* was a constant = 1.96; *P* is a known or estimated prevalence; *Q* = (1 − P); *L* is the allowable error. As the prevalence of BTV in different regions of the Gadarif State has never been substantially determined, this study assumed an expected prevalence of BTV in domestic ruminants to be 50%. The number of animals estimated using this was 384. A total number of 1938 serum samples (420 cattle, 877 sheep, and 641 goats) were included in this survey.

The study received ethical approval from the ethical committee of the Central Veterinary Research Laboratory (CVRL), Animal Resources Research Corporation (ARRC).

### 2.3. Collection of Blood Samples

Sample collection was carried out according to the animal welfare code of Sudan. The collection of blood from animals was implemented by competent veterinarians following appropriate physical control of animals to confirm both personnel and animal protection. Blood samples (*n* = 1938; 420 cattle, 877 sheep, and 641 goats) were collected randomly from apparently healthy animals during the period 2015-2016. Serum was separated by centrifugation and frozen at −20°C until tested. Risk factors analyzed in this study comprised age, sex, breed (cattle: Butana, Gash, Cross and Kenana; goat: Nubian and Cross), ecology (desert, semiarid, savanna, and rich savanna), and locality. It worth mentioning that BTV vaccination is not practiced in Sudan.

### 2.4. Enzyme-Linked Immunosorbent Assay (ELISA)

An indirect enzyme-linked immunosorbent assay (cELISA) was conducted using commercially available BTV kits for the detection of specific antibodies to BTV VP7 protein (Ingezim, Spain) according to the manufacturer's instructions. Briefly, ELISA was performed in 96 well antigen coated microplates. The incubation was performed for 30 min at room temperature, and the plates were then washed six times. The conjugate was added and incubated for 45 min, and the substrate was added and incubated for 10 min. The reaction was stopped using stop solution, and then results were read using an ELISA reader (Biochrome, England) set at 630 nm. The sensitivity and specificity of ELISA used in this study were 100% and 99.8%, respectively [11].

### 2.5. Statistical Analysis

Statistical package for Social Science (SPSS) software (version 16.0) was employed for the results' analysis. Association between the outcomes variables and its risk factors was firstly screened in univariate analysis. Then, the multivariable model for the outcome variable was constructed, and BTV infection was considered as a dependent variable and the risk factors as independent variables. Finally, odd ratios 95% at the confidence interval were calculated. A *p* value of ≤0.05 indicated a significant association.

## 3. Results

Univariate analysis revealed a significant difference (*p* < 0.05) between location, age, area ecology, and seropositivity to BTV in all animal species besides sex in sheep (Tables 1, 2, 3). Using multivariate analysis, age, location, and area ecology were the main risk factors for BTV seropositivity in all animal species tested. In addition, the overall seroprevalence rate of BTV was 92.9% (390/420) in cattle. The surveyed cattle breeds' revealed the presence of the highest BTV antibodies in Kenana (96.8%) while the lowest in Butana (89.9%) with insignificant differences (Table 4). Only three independent risk factors were statistically significant. Older cattle (>48 months) were four times more likely to be seropositive with BT (OR = 4.95, CI = 1.46–16.78, *p* value = 0.01), cattle sampled from Gla Nahal were eight times more probable to be seropositive (OR = 8.67, CI = 1.01–74.72, *p* value = 0.049), and animals in semiarid areas were seven times more likely suitable for BTV seropositivity (OR = 7.719, CI = 1.794–33.211, *p* value = 0.006) (Table 4). The overall seroprevalence rate in sheep was 76.4% being the highest in Algoraisha and Galabat west with 84.8% and 83.7% prevalence rate, respectively, while the lowest in Butana and Gadarif center being 63.3% and 66.3%, respectively. There were significant differences (*p* < 0.05) between location, sex, area ecology, age groups, and seropositivity to BTV. Older sheep (>24 months) were twice as likely to be seropositive with BTV (OR = 2.46, CI = 1.49–4.07, *p* value = 0.0001), females were approximately twice as susceptible for BTV seropositivity (OR = 1.999, CI = 1.38–2.89, *p* value = 0.0001), and sheep in the rich savanna area were twice as susceptible for BTV seropositivity (OR = 2.32, CI = 1.36 – 3.95, *p* value = 0.002) (Table 5). The overall seroprevalence rate was 85.3% in goats. Similarly, there was a significant difference (*p* < 0.05) between location, area ecology, age groups, and seropositivity to BTV. Older goats (>24 months) were four times more likely to be seropositive (OR = 4.93, CI = 2.67–9.12, *p* value = 0.0001). Poor savanna animals were eight times more likely suitable for BTV seropositivity (OR = 8.82, CI = 4.66–16.70, *p* value = 0.0001) (Table 6). Sheep sampled from Basonda, Gla Nahal, Alrahad Algoraisha, Baladiat Gadarif, and Glabat west locations were two to three times more likely to be seropositive (OR = 3.23, CI = 1.58–6.58, *p* value = 0.001) (Table 2), whereas goats sampled from Algoraisha, Baladiat Gadarif, Alfashaga, Galabat west, and Fao locations were six to nine times more likely to be seropositive (OR = 9.08, CI = 4.06–20.31, *p* value = 0.0001) (Table 6).

## 4. Discussion

The overall seroprevalence rate of BTV in cattle was 92.8%, which is similar to findings in Iran (93.5%) and Turkey (88%), but higher than that reported in cattle in Saudi Arabia (44%), Algeria (28%), Egypt (51.47%), Pakistan (52%), Italy (43.6%) [12–17], and in previous surveys in Sudan [6–8, 18]. These differences could be attributed to the testing method used, type, and size of samples tested. The overall BTV seroprevalence in sheep and goats in Gadarif State was found to be 76.4% and 85.3%, respectively. It is however higher than in Ethiopia [19] and Saudi Arabia [20]. The high seroprevalence in goats resembles that reported by Elmahi [21] in Kassala State. A recent finding indicates that goats may play a critical role in BTV epidemiology, as goats are more resistant and can survive the infection [22]. Moreover, goats with their minimum clinical manifestation keep higher titers of BTV and probably the possible origin of infection to new generation of susceptible animals. This may be the case for goats that exhibited very high seropositivity in both states calling for further in depth investigations. The significant association between BTV seropositivity and the increasing age of cattle, sheep, and goats may be due to frequent exposure of older animals to the competent vector. This is in agreement with previous epidemiological surveys conducted in Sudan [5, 8]. The BTV-specific antibodies detected among cattle suggested a natural infection as BTV vaccination is not practiced. Furthermore, the cattle tested were older than 6 months; therefore, maternal antibodies were no longer present [23]. But BTV-specific antibodies detected among sheep and goats aged 1–6 months may be due to maternal antibodies. A significant association was noted between BTV seropositivity and sampled cattle from Gla Nahal location. This might be attributed to the heavy rainfall and subsequent higher abundance of the vector. In cattle, a significant association (*p* < 0.05) was observed between BTV infection and semiarid ecology, which is not adequate to support large populations of *Culicoides* midges (temperature 30−45°C, low humidity, and precipitation below potential evapotranspiration); this could be interpreted by existence of foci around seasonal water bonds suitable for Culicoides breeding, or by movement in and out of the areas. Or else, other ways of acquiring the infection like contact and transplacental transmission might be occurring in this area [24, 25], matter that needs to be verified. Generally, the high seroprevalence seen herein is in line with the findings reported by Elmahi and Elhassan et al. [5, 18] in Sudan. Indeed, it is now notable that cattle play a key role in BTV epidemiology. Cattle may serve as a source of virus for several weeks while displaying little or no clinical signs of disease and are often the preferred host for insect vectors [26]. Further surveillance for BTV to evaluate this evidence among cattle in Sudan is needed. Also, area ecology showed a significant difference (*p* < 0.05) in seroprevalence rates of BTV infection in sheep and goats. This may be due to the fact that BTV infections have association with rainfall estimates and ecological categories such as savanna and rich savanna [27]. Further, the BTV seroprevalence rate in sheep was 65.2% in males, while it was 79.0% in females which agrees with Elmahi [21]. This could have been explained by the fact that females are not consumed for meat and kept for breeding, whereas males are slaughtered at younger age.

## 5. Conclusions

It could be concluded that BTV antibodies are highly prevalent in Gadarif State, Sudan. Thus, these animals are protected against certain BTV serotypes. A strategy to control Bluetongue disease and which serotypes are circulating in the area is thus urgently recommended.

## Figures and Tables

**Table 1:** Univariate analysis for the association between potential risk factors and BTV seropositivity among cattle in Gadarif State, Eastern Sudan.

Risk factor	Animals tested	Animals affected (%)	*p* value
Location			
Mafaza	35	27 (77.1)	0.001
GlaNahal	31	30 (96.8)	
Glabat north	46	41 (89.1)	
Butana	35	35 (100)	
Gadarif center	36	36 (100)	
Alrahad	31	29 (93.5)	
Algoraisha	35	29 (82.9)	
BaladiatGadarif	33	31 (93.9)	
Alfashaga	46	46 (100)	
Glabat west	46	42 (91.3)	
Fao	46	44 (95.7)	
Sex			
Male	130	120 (92.3)	0.770
Female	290	270 (93.1)	
Age (months)			
6–11	226	202 (89.4)	0.044
12–24	24	22 (91.7)	
24–36	21	20 (95.2)	
36–48	15	15 (100)	
>48	128	125 (97.7)	
Breed			
Butana	148	133 (89.9)	0.203
Gash	156	149 (95.5)	
Cross	81	74 (91.4)	
Kenana	31	30 (96.8)	
Ecology			
Desert	35	35 (100%)	0.001
Semiarid	115	113 (98.3%)	
Savanna	208	183 (88.0%)	
Rich savanna	62	59 (95.2%)	

**Table 2:** Univariate analysis for the association between potential risk factors and BTV seropositivity among sheep in Gadarif State, Eastern Sudan.

Risk factor	Animals tested	Animals affected (%)	*p* value
Location			
Butana	90	57 (63.3)	0.003
Basonda	51	42 (82.4)	
GlaNahal	92	72 (78.3)	
Fao	92	64 (69.6)	
Gadarif center	92	61 (66.3)	
Alrahad	92	74 (80.4)	
Algoraisha	92	78 (84.8)	
BaladiatGadarif	92	75 (81.5)	
Alfashaga	92	70 (76.1)	
Glabat west	92	77 (83.7)	
Sex			
Male	164	107 (65.2)	0.0001
Female	713	563 (79.0)	
Age (months)			
1–6	108	72 (66.7)	0.001
6–12	149	104 (69.8)	
12–24	330	253 (76.7)	
>24	290	241 (83.1)	
Ecology			
Desert	90	57 (63.3)	0.007
Semiarid	276	206 (74.6)	
Savanna	276	219 (79.3)	
Rich savanna	235	188 (80.0)	

**Table 3:** Univariate analysis for the association between potential risk factors and BTV seropositivity among goats in Gadarif State, Eastern Sudan.

Risk factor	Animals tested	Animals affected (%)	*p* value
Location			
Butana	90	46 (51.1)	0.0001
Alrahad	92	92 (100)	
Algoraisha	92	82 (89.1)	
BaladiatGadarif	92	83 (90.2)	
Alfashaga	92	83 (90.2)	
Galabat west	92	79 (85.9)	
Fao	92	83 (90.2)	
Sex			
Male	78	65 (83.3)	0.594
Female	563	482 (85.6)	
Age (months)			
1–6	82	56 (68.3)	0.0001
6–12	41	29 (70.7)	
12–24	217	187 (86.2)	
>24	302	276 (91.4)	
Breed			
Nubian	610	519 (85.1)	0.421
Cross	31	28 (90.3)	
Ecology			
Desert	90	46 (51.1)	0.0001
Semiarid	184	166 (90.2)	
Savanna	276	244 (88.4)	
Rich savanna	92	92 (100)	

**Table 4:** Multivariate analysis for the association between potential risk factors and BTV seropositivity among cattle in Gadarif State, Eastern Sudan.

Risk factor	Animals tested	Animals affected (%)	*p* value	Odds ratio	95% CI lower-upper
Location					
Mafaza	35	27 (77.1)	Ref	—	—
Gla Nahal	31	30 (96.8)	0.049	8.667	1.005–74.724
Glabat north	46	41 (89.1)	0.851	0.866	0.193–3.891
Butana	35	35 (100)	0.998	—	—
Gadarif center	36	36 (100)	0.998	—	—
Alrahad	31	29 (93.5)	0.080	4.314	0.840–22.156
Algoraisha	35	29 (82.9)	0.125	0.073	0.003–2.056
Baladiat Gadarif	33	31 (93.9)	0.426	0.330	0.022–5.062
Alfashaga	46	46 (100)	0.997	—	—
Glabat west	46	42 (91.3)	0.618	0.556	0.055–5.590
Fao	46	44 (95.7)	0.129	3.580	0.688–18.616
Sex				1.103	0.474–2.570
Male	130	120 (92.3)	Ref		
Female	290	270 (93.1)	0.820		
Age (months)					
6–11	226	202 (89.4)	Ref	—	—
12–24	24	22 (91.7)	0.728	1.307	0.289–5.905
24–36	21	20 (95.2)	0.409	2.376	0.305–18.504
36–48	15	15 (100)	0.999	—	—
>48	128	125 (97.7)	0.010	4.950	1.460–16.781
Breed					
Butana	148	133 (89.9)	Ref	—	—
Gash	156	149 (95.5)	0.064	2.401	0.950–6.067
Cross	81	74 (91.4)	0.714	1.192	0.465–3.056
Kenana	31	30 (96.8)	0.247	3.383	0.430–26.617
Ecology					
Desert	35	35 (100%)	0.998	—	—
Semiarid	115	113 (98.3%)	0.006	7.719	1.794–33.211
Savanna	208	183 (88.0%)	Ref	—	—
Rich savanna	62	59 (95.2%)	0.116	2.687	0.783–9.219

**Table 5:** Multivariate analysis for the association between potential risk factors and BTV seropositivity among sheep in Gadarif State, Eastern Sudan.

Risk factor	Animals tested	Animals affected (%)	*p* value	Odds ratio	95% CI lower-upper
Location					
Butana	90	57 (63.3)	Ref	—	—
Basonda	51	42 (82.4)	0.028	2.084	1.082–4.013
Gla Nahal	92	72 (78.3)	0.020	2.702	1.169–6.245
Fao	92	64 (69.6)	0.374	1.323	0.714–2.453
Gadarif center	92	61 (66.3)	0.675	1.139	0.620–2.094
Alrahad	92	74 (80.4)	0.011	2.380	1.218–4.652
Algoraisha	92	78 (84.8)	0.001	3.226	1.582–6.576
Baladiat Gadarif	92	75 (81.5)	0.007	2.554	1.295–5.036
Alfashaga	92	70 (76.1)	0.063	1.842	0.969–3.504
Glabat west	92	77 (83.7)	0.002	2.972	1.476–5.984
Sex					
Male	164	107 (65.2)	Ref	—	—
Female	713	563 (79.0)	0.0001	1.999	1.383 – 2.890
Age (months)					
1–6	108	72 (66.7)	Ref	—	—
6–12	149	104 (69.8)	0.594	1.156	0.679–1.966
12–24	330	253 (76.7)	0.040	1.643	1.022–2.640
>24	290	241 (83.1)	0.0001	2.459	1.485–4.072
Ecology					
Desert	90	57 (63.3)	Ref	—	—
Semiarid	276	206 (74.6)	0.04	1.704	1.026–2.830
Savanna	276	219 (79.3)	0.003	2.224	1.325–3.735
Rich savanna	235	188 (80.0)	0.002	2.316	1.357–3.953

**Table 6:** Multivariate analysis for the association between potential risk factors and BTV seropositivity among goats in Gadarif State, Eastern Sudan.

Risk factor	Animals tested	Animals affected (%)	*p* value	Odds ratio	95% CI lower-upper
Location					
Butana	90	46 (51.1)	Ref	—	—
Alrahad	92	92 (100)	0.996	—	—
Algoraisha	92	82 (89.1)	0.0001	8.465	3.859–18.567
Baladiat Gadarif	92	83 (90.2)	0.0001	8.692	3.796–19.902
Alfashaga	92	83 (90.2)	0.0001	9.082	4.061–20.313
Galabat west	92	79 (85.9)	0.0001	6.207	2.970–12.975
Fao	92	83 (90.2)	0.0001	9.050	4.047–20.241
Sex					
Male	78	65 (83.3)	Ref	—	—
Female	563	482 (85.6)	0.191	1.603	0.790–3.254
Age (months)					
1–6	82	56 (68.3)	Ref	—	—
6–12	41	29 (70.7)	0.783	1.122	0.495–2.542
12–24	217	187 (86.2)	0.001	2.894	1.582–5.295
>24	302	276 (91.4)	0.0001	4.929	2.665–9.115
Breed					
Nubian	610	519 (85.1)	Ref	—	—
Cross	31	28 (90.3)	0.425	1.636	0.487–5.495
Ecology					
Desert	90	46 (51.1)	Ref	—	—
Semiarid	184	166 (90.2)	0.0001	8.821	4.659–16.70
Savanna	276	244 (88.4)	0.0001	7.293	4.192–12.68
Rich savanna	92	92 (100)	0.996	—	—

## Data Availability

The data used to support the findings of this study are available from the corresponding author upon request.
